# Modulating immunometabolism in transition dairy cows: the role of inflammatory lipid mediators

**DOI:** 10.1093/af/vfac062

**Published:** 2022-10-14

**Authors:** Maya Zachut, Joseph Tam, Genaro Andres Contreras

**Affiliations:** Department of Ruminant Science, Institute of Animal Sciences, Agriculture Research Organization Volcani Institute, Rishon LeZion, Israel; Obesity and Metabolism Laboratory, Institute for Drug Research, School of Pharmacy, Faculty of Medicine, The Hebrew University of Jerusalem, Jerusalem, Israel; Department of Large Animal Clinical Sciences, Michigan State University, East Lansing, MI, USA

**Keywords:** endocannabinoids, fatty acids, immune, niacin, oxylipid, transition dairy cow

**Implications**

- Successful transition to lactation requires strong immune function that is supported by adequate function of metabolic organs.
- Immunometabolism is the connection between immune function and metabolism. Transition cows with well-regulated immunometabolic signals can better modulate inflammatory responses.
- During un-balanced immunometabolic response, signals from metabolic tissues can affect immune cells’ function and promote inflammation.
- Lipid mediators such as endocannabinoids, fatty acids, and oxylipids are potent modulators of immunometabolic functions.
- Dietary supplementation of omega-3 and conjugated linoleic fatty acids, and inhibitors of lipolysis (i.e., fatty acid release), such as niacin, can effectively modulate immunometabolism in transition dairy cows via lipid mediators.

## Introduction

Immunometabolism describes the integration of nutrient sensing, immune signaling, and immune cell metabolism. One of the basic concepts of immunometabolism is that fine-tuning of nutrient supply can modulate specific metabolic pathways in immune cells. Immune cells’ phenotypes are associated with distinct metabolic configurations: resting immune cells rely on energetically efficient metabolic processes, such as the tricarboxylic acid (TCA) cycle, while activated immune cells shift to glycolysis (Pearce and Pearce, 2013). Thus, changes in immune cell function are characterized not only by alterations in their inflammatory phenotype but also by changes in their metabolism (Pearce and Pearce, 2013). In recent years, immunometabolism research raised the concept that during inflammation or immune activation, it is possible to manipulate metabolism using small molecules and metabolic intermediates, termed here as “immunomodulators,” that may affect immune cell response (Palsson-McDermott, 2015). At the same time, since immune cells are potent modulators of systemic metabolism, altering immune cell function will shift metabolic function in metabolic organs.

In dairy cows, the transition from late pregnancy to the early postpartum (PP) period and the onset of lactation is characterized by vast changes in metabolism and immune function, thus the concept of immunometabolism is particularly relevant at this stage of the dairy cows’ life. During the first weeks PP, as part of the physiological adaptation to parturition, cows exhibit a variable degree of systemic subacute inflammation, which involves a mild increase in pro-inflammatory mediators such as cytokines and acute phase proteins (Bradford and Swartz, 2020). Inflammation is necessary for periparturient processes such as placental expulsion and uterine involution, and it is usually well regulated to avoid excessive damage to the host. However, when dysregulated, inflammation can cause irreparable damage to tissues, and may lead to disease. In addition, PP cows experience dysregulation in lymphocyte and neutrophil function (Contreras and Sordillo, 2011). The PP period is also characterized by increased oxidative stress that is driven by the imbalance between the production of reactive oxygen species (ROS), reactive nitrogen species, and the neutralizing capacity of antioxidant mechanisms in tissues and blood. Increased oxidative stress can affect immune function; for example, in adipose tissue increased ROS can enhance the release of pro-inflammatory cytokines and thus maintain the inflammatory processes (Lavrovsky et al., 2000). Thus, regulating immunometabolism in periparturient cows is important for a successful transition period and lactation.

### Periparturient drivers of changes in immunometabolism

**Figure 1.**
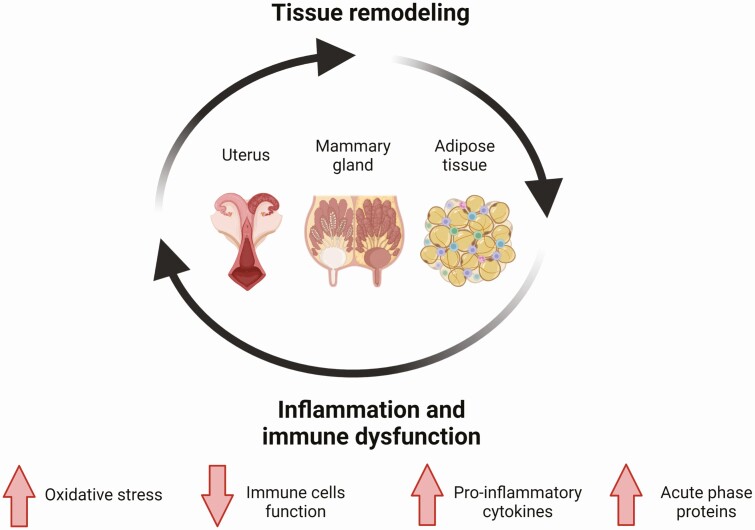
Tissue remodeling drivers of immunometabolism in transition dairy cows. Following parturition, uterine tissues are remodeled and local inflammation occurs; lactation leads to remodeling of the mammary gland for milk production, and negative energy balance promotes adipose tissue lipolysis and remodeling that involves an inflammatory response. These remodeling processes drive immunometabolic changes in the transition dairy cow, including systemic subacute inflammation that is characterized by elevated pro-inflammatory cytokines and acute phase proteins, increased oxidative stress, and immune cell dysregulation. These changes can further promote tissue inflammation, leading to immunometabolic shift. Image was prepared using Biorender.com.

Inflammatory processes common to the periparturient period, such as placental expulsion, uterine involution, lipid mobilization, and lactogenesis are driven by tissue remodeling (Contreras et al., 2018). For example, lipolysis is a potent promotor of inflammation in adipose tissues and systemically. Tissue remodeling and accompanied tissue damage could have an important role in immunometabolism of transition dairy cows, as they attract cells of the innate immune system to tissues with active inflammatory processes, and may facilitate the penetration of microorganisms in these organs, which would lead to an invasion of immune cells that produce pro-inflammatory cytokines (Kuhla, 2020). Inflammatory mediators can influence nutrient influx and partitioning in metabolic organs; it was proposed by Bradford and Swartz (2020) that inflammatory mediators, working in part through altered insulin sensitivity, may underlie some of the variations in the rate of tissue catabolism between cows during the early PP period (Zachut, 2015), which is consistent with the emerging roles of immune cells and signals in regulating adipose tissue metabolism. Another important aspect of immunometabolism in transition cows is the use of glucose as a source of energy for immune cells. After parturition, circulating glucose is prioritized to the non-insulin-dependent glucose transporters, which are expressed mainly in immune cells and the mammary gland (Lopreiato et al., 2020). The large glucose requirements of an activated immune system during systemic inflammation could further reduce the energy available for the mammary gland, aggravating the negative energy balance (Kvidera et al., 2017). Figure 1 summarizes the main tissue remodeling processes that are involved in immunometabolism of transition dairy cows.

## Lipid-derived Immunomodulators

### Endocannabinoids

The endocannabinoid system (ECS) is a central regulator of metabolism and energy homeostasis (Silvestri and Di Marzo, 2013), that is also involved in immune modulation in mammals. The ECS components are the ligands, receptors, and enzymes that synthesize and degrade the endogenous ligands of the ECS, called endocannabinoids (eCBs). Detailed information on the components of the ECS and their metabolic effects and proposed role in dairy cows was recently reviewed (Myers et al., 2021), and readers can find detailed mechanistic details on the ECS therein. In short, eCBs are lipid intermediaries that include amides, esters, and ethers of polyunsaturated fatty acids (FAs). They are quickly synthesized from FAs in response to intracellular calcium influx, metabolic stress, or cellular damage. Arachidonic acid (AA)-derived eCBs: *N*-arachidonoylethanolamide (AEA or anandamide) and 2-arachidonyglycerol (2-AG) are the most abundant and well characterized, although there are other eCB-like molecules, such as *N*-palmitoylethanolamine (PEA) and *N*-oleoylethanolamine (OEA), which derived from palmitic and oleic acids, respectively. eCBs primarily bind and activate the cannabinoid receptors type 1 (CB1) and 2 (CB2), although they can also bind to receptors such as the vallinoid receptor TRPV1, G protein-coupled receptor 55 (GPR55), and members of the peroxisome proliferator-activated receptor (PPAR) family. The CB1 is prevalently localized in the central nervous system and in peripheral tissues, while CB2 is mainly expressed by immune cells (Myers et al., 2021). Within the scope of the present review, we will focus on the possible involvement of the ECS in inflammation and immune regulation.

In mammals, eCBs participate in the fine regulation of homeostasis of the immune system, exerting mainly anti-inflammatory and immunosuppressive effects mediated by the stimulation of CB2. CB2 activation influences different functional aspects of immune cells, such as migration, proliferation, cell death, and secretion of cytokines (Basu and Dittel, 2011). Therefore, eCBs can be considered as immunomodulators that may constitute a new class of anti-inflammatory molecules. The eCB AEA has anti-inflammatory effects, such as the inhibition of chemoattractant cytokines secretion; while AA and 2-AG can exert pro-inflammatory effects; and, inhibition of ECS activity may cause inflammatory responses (Myers et al., 2021). Therefore, more research is required to fully elucidate the effects of eCBs as immunomodulators in different tissues and physiological conditions.

**Figure 2.**
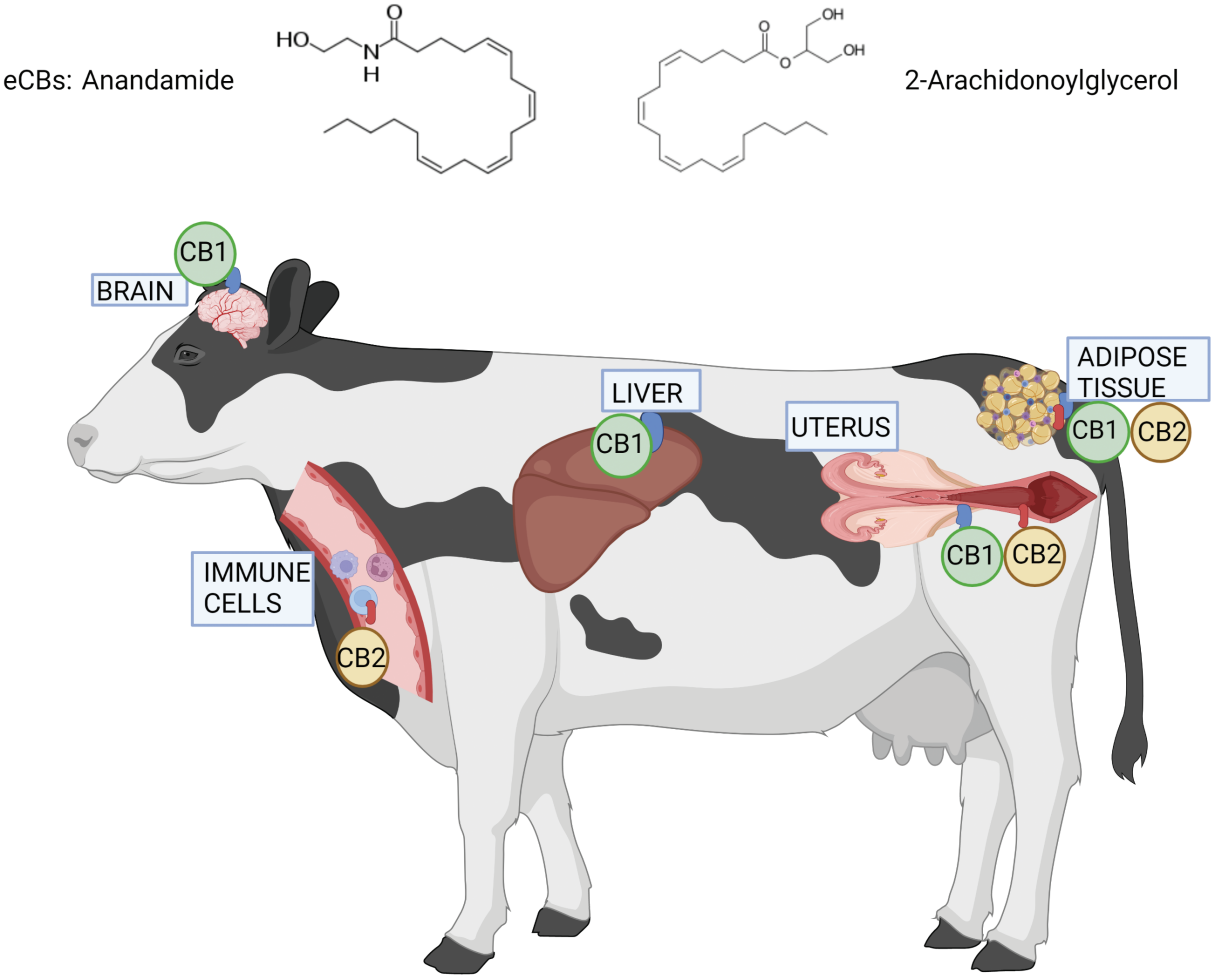
Model of ECS components in immunometabolic tissues in transition dairy cows. The brain and peripheral tissues such as the liver, adipose tissue, and uterus express the cannabinoid receptor type 1 (CB1), while the cannabinoid receptor type 2 (CB2) is expressed in immune cells that are localized in the blood and may also be present in peripheral tissues. The ligands of the ECS, the eCBs, such as anandamide and 2-arachidonoylglycerol, can bind to these receptors and activate the ECS, which has immunometabolic effects. Image was prepared using Biorender.com.

In transition dairy cows, levels of AEA and 2-AG in adipose tissue were doubled PP relative to prepartum, and it was shown that cows that had a higher degree of body weight loss PP had higher levels of eCBs in their adipose relative to cows with less body weight loss (Zachut et al., 2018). Plasma 2-AG concentrations were found to be low during the dry period and then increased PP (Kuhla et al., 2020). Higher gene expression of CNR2/CB2 and the ECS enzymes *N*-acyl phosphatidylethanolamine phospholipase D (NAPEPLD) and fatty acid amide hydrolase (FAAH), which synthesize and degrade AEA, respectively, in adipose tissue was related to increased expression of genes of pro-inflammatory cytokines (TNF-α, IL-6, IL-1β) at 21 and 42 d PP in cows with intense lipolysis (Dirandeh et al., 2020). Thus, the ECS could be involved in adipose tissue lipolysis and inflammation in PP cows. Activation of the ECS may also be related to subclinical inflammatory diseases, as periparturient cows with subclinical endometritis had decreased expression of the eCBs hydrolyzing enzymes *N*-acylethanolamine acid amidase (*NAAA*) and *FAAH*, and increased expression of *NAPEPLD* and CNR2/CB2 in uterus compared to controls (Bonsale et al., 2018). Together with the findings in adipose, this may suggest a possible link between the ECS and inflammatory responses in transition cows; however, more research elucidating the connection between the two in transition dairy cows is still required. Figure 2 presents a proposed model of ECS components in immunometabolic tissues of transition dairy cows.

### Fatty acids and oxylipids

Fatty acids released from phospholipids and lipid droplets (especially in adipocytes and macrophages) are potent modulators of immune and metabolic functions. Periparturient lipolysis in adipose tissues increases the release of lipids into circulation, and therefore, the abundance of specific FAs in plasma, that can activate the PPAR α, γ, β/δ in several types of cells (Contreras et al., 2017). In macrophages, PPARγ activation defines their phenotype by modulating the maturation of monocytes and pre-macrophages in tissues (Toobian et al., 2021). During inflammatory processes, PPARγ activity inhibits the production of pro-inflammatory cytokines and promotes immunotolerance (Chawla, 2010). In contrast, PPARα appears to promote early inflammatory responses in macrophages and reduce immunotolerance (Toobian et al., 2021). FAs can also modulate the metabolism of immune cells through their oxidized products known as oxylipids (also termed oxylipins). Upon release by lipases from phospholipid membranes or lipid droplets, polyunsaturated FAs are rapidly metabolized and may be oxidized to produce oxylipids by enzymatic and non-enzymatic reactions (Sordillo, 2018). Linoleic acid is the most abundant polyunsaturated FA in adipose tissue and is preferentially mobilized during the transition period (Contreras et al., 2017). In immune cells, phospholipases preferentially release AA from the cellular membrane. Therefore, the most abundant oxylipids in dairy cattle are those derived from linoleic and arachidonic acids. Oxylipids are potent lipid mediators of inflammation that link immune function and metabolism in several ways including the activation of nuclear receptors, mitochondrial activity and biogenesis, and the modulation of lipid mobilization in adipose, liver, and immune cells. Importantly, final disposition of oxylipids occurs through mitochondrial β-oxidation (Misheva et al., 2022), demonstrating a paramount connection between the inflammatory process and metabolic function within cells.

Linoleic acid is highly susceptible to oxidation by 5- and 15-lipoxygenases (LOX) and by non-enzymatic reactions triggered by ROS to produce hydroxyoctadecadienoic acids (HODEs). These oxylipids, including 9-, 10-, 12-, and 13-HODE trigger immunometabolic responses by binding to their cellular membrane receptors GPR132 and TLR4 (Obinata et al., 2005). As immunometabolic messengers, HODEs functions vary by regioisomer. For example, 13-HODE, a product of 15-LOX, promotes M2 polarization during lipolysis and acts as a PPARγ ligand that promotes adipogenesis and lipogenesis (Lee et al., 2016). In healthy transition cows, plasma 13-HODE increases at 1 wk after calving from its levels at 1 wk before parturition (Contreras et al., 2017). In adipose, 9-HODE tends to increase after parturition and 13-HODE is higher than at either 1 or 4 wk before calving. Adipose content of 13-HODE was positively associated with plasma beta-hydroxybutyrate concentrations.

**Figure 3.**
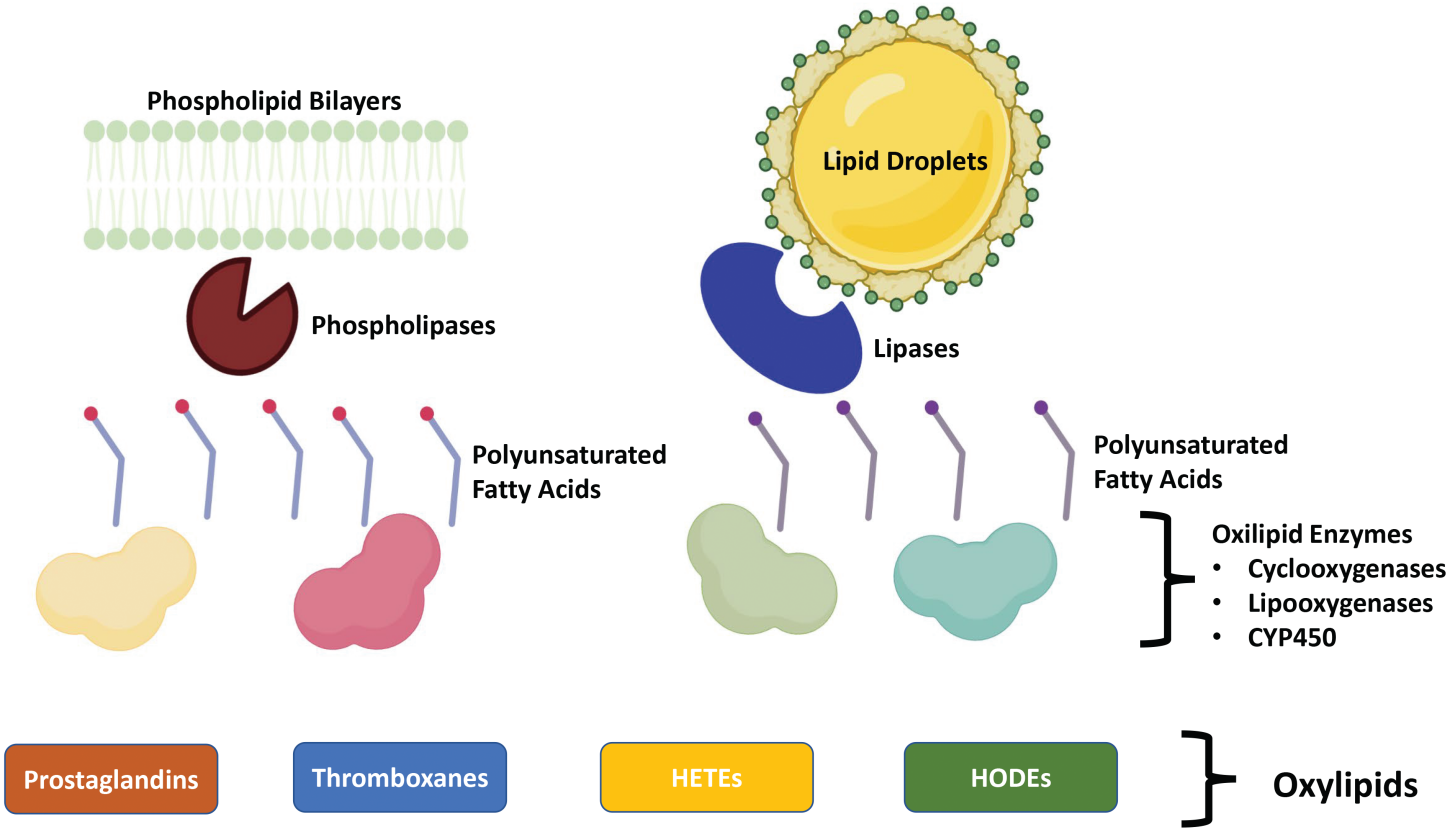
General pathways for oxylipid biosynthesis. During inflammatory processes, polyunsaturated fatty acids are released from lipid stores, such as lipid droplets and phospholipid bilayers by phospholipases and lipases (e.g., hormone-sensitive lipase). Polyunsaturated fatty acids are then oxidized by cyclooxygenases, lipooxygenases, and cytochrome P450 (CYP450) enzymes to produce oxylipids. Among these, the immunometabolic effects of prostaglandins, thromboxanes, hydroxyeicosatetraenoic (HETE), and hydroxyoctadecadienoic (HODE) acids are characterized in transition dairy cows. Image was prepared using Biorender.com.

Arachidonic acid is oxygenated by enzymes and by non-enzymatic reactions to produce prostaglandins (PGs), thromboxanes (TXs), and hydroxyeicosatetraenoic acids (HETEs). PGs and TXs are modulators of different stages of the inflammatory process (Sordillo, 2018). PGs also modulate metabolic pathways in immune cells and support cellular phenotype polarization, especially in macrophages (Saha et al., 2017). In healthy transition cows, plasma content of 5-HETE and 20-HETE increases during the first week after calving compared to prepartum (Contreras et al., 2017). As reviewed in detail in Sordillo et al. (2008), 5-HETE modulates macrophage activity by enhancing their phagocytosis capacity, it enhances lymphocyte proliferation and is a strong chemoattractant for bovine neutrophils. In addition, 15-HETE and its precursor 15-hydroperoxyoctadecadienoic acid are potent inducers of adhesion molecule expression in endothelial cells; therefore, regulating neutrophil and mononuclear cell migration into tissues (Sordillo et al., 2008). Figure 3 summarizes the general pathways of oxylipid biosynthesis during inflammation and the immunomodulatory lipid mediators that are characterized in transition dairy cows. Together, FAs and oxylipids are prominent immunomodulators in PP cows. Interestingly, there is strong evidence of the combined effects of FAs, oxylipids, and eCBs in regulation of inflammation (de Bus et al., 2019), and this interaction between lipid mediators should be explored in dairy cows.

## Dietary Immunomodulators in Transition Dairy Cows

### Omega-3 fatty acids

Dietary supplementation of omega-3 FAs has positive effects on the performance of dairy cows (Moallem, 2018). Across species, omega-3 FAs are known to have anti-inflammatory effects that are mediated via altered cytokine secretion and/or PPARγ; as well as by eicosanoid, resolving, and protectin biology. Long-chain omega-3 consumption promotes the incorporation of eicosapentaenoic acid (EPA) into membrane phospholipids at the expense of AA (Grimm et al., 2002). Wherein, EPA competitively inhibits the conversion of AA into potent oxylipids, such as prostaglandin E2 and leukotriene B4. As an alternative substrate, EPA-derived cyclooxygenase (COX) and lipoxygenase (LOX) enzyme products, such as prostaglandin E3 and leukotriene B5, are much less potent (Grimm et al., 2002). Furthermore, resolvins are another group of oxylipids, derived from docosahexaenoic acid (DHA), which promote the resolution of inflammation. In bovine cells, omega-3 FAs were shown to have anti-inflammatory effects in endothelial cells (Contreras et al., 2012), and to lower the lymphocyte response to mitogens and natural killer cell ability (Worden et al., 2018).

In transition dairy cows, there is evidence of moderate anti-inflammatory effects of omega-3 supplementation. Silvestre et al. (2011) showed that feeding calcium salts of fish oil (rich in long-chain omega-3, EPA, and DHA) attenuated the production of the pro-inflammatory cytokine TNF-α in neutrophils that were challenged with lipopolysaccharide (LPS). Transition cows supplemented with omega-3 from algae (rich in EPA and DHA) and vitamin E showed a milder inflammatory response compared to controls, as demonstrated by lower levels of bilirubin and by a higher level of liver functionality index (Trevisi et al., 2011), however, another study reported that omega-3 from fish oil did not affect systemic markers of inflammation (Grossi et al., 2013). The peripheral blood mononuclear cells (PBMC) of cows supplemented with omega-3 from flaxseed (rich in alpha-linolenic acid [ALA]) had higher abundance of the p65-subunit-of-transcription-factor NF-κB, whereas albumin, C4b-binding protein, and complement factor H levels were lower compared to controls, and expression of NF-κB in white blood cells was lower than in controls (Kra et al., 2021). Supplementing transition cows with omega-3 can also modulate the biosynthesis of the immunometabolic modulators—oxylipids (Sordillo, 2018) and eCBs (Zachut, 2020). Dirandeh and Ghaffari (2018) fed dairy cows with omega-3 from flaxseed (rich in ALA) from calving and collected uterine biopsies at the day of ovulation; they found that omega-3 supplementation increased endometrial expression of the ECS enzymes NAAA and monoglyceride lipase (MGLL), while the expression of CNR2/CB2 and the ECS enzyme NAPEPLD were decreased in cows fed omega-3 compared to controls. Recently, we demonstrated that supplementing transition cows with omega-3 from flaxseed altered ECS components in the blood, adipose tissue, and liver, and this was associated with moderate modulations in lipid metabolism in the adipose and inflammation in the liver (Kra et al., 2022). These studies suggest that supplementation of omega-3 is a strategy to modulate the activation of the ECS in dairy cows. The immunometabolic effects of long-chain omega-3 (EPA and DHA) may be mediated mainly via resolvins, while supplementing high proportions of ALA can profoundly lower the omega-6/omega-3 ratio in the diet, consequently affecting immunometabolism also via the ECS.

### Conjugated linoleic acid

Supplementation of conjugated linoleic acid (CLA) induces milk fat depression in dairy cows (Jenkins and Harvatine, 2014). In PP cows, feeding CLA can improve their metabolic state by decreasing energy output in milk. At the same time, there is evidence that CLA act as a lipid immunomodulator in transition dairy cows. The energy-conserving effect of CLA may reduce adipose lipolysis PP, which would possibly decrease inflammation. Moreover, the conjugated isomers of linoleic acid, *cis*-9, *trans*-11 and *trans*-10, *cis*-12, may have anti-inflammatory effects via interaction with PPARγ (Lee et al., 2010). In a comprehensive study with transition dairy cows, the effects of essential FAs (EFAs) and CLA on metabolic and inflammatory indices were examined; supplementation of a combination of EFAs and CLA improved the metabolic state of the cows, increased their energy balance, and had a moderate effect on hepatic acute phase proteins, while the effect of CLA alone on inflammatory markers was minor (Gnott et al., 2020). The effects of CLA supplementation were also evaluated in adipose tissues and liver; in adipose tissues, it was found that CLA supplementation affected the integrated phosphoproteome toward phosphorylation of proteins related to lipolysis and lipogenesis (Daddam et al., 2021). In the liver of cows supplemented with a combination of CLA and EFAs, the relative abundance of several proteins related to CYP450 epoxidation/hydroxylation was affected (Veshkini et al., 2022), and this was proposed to be related to an immunometabolic effect of CLA, as CYPP450 regulates the cross-talk between the immune system and metabolism. In another study, Gross et al. (2018) demonstrated that in early lactating cows that were exposed to an intramammary LPS challenge, CLA supplementation affected local and systemic immune responses. An in vitro experiment demonstrated that a 50:50 (*cis*-9,*trans*-11:*trans*-10,*cis*-12) CLA mixture reduced monocyte apoptosis and increased the extracellular respiratory burst, suggesting that CLA isomers do have immunomodulatory effects on some functions of bovine monocytes (Ávila et al., 2020). Taken together, it seems that CLA supplementation may have a moderate immunometabolic effect in transition dairy cows.

Supplementation of CLA to transition dairy cows can also affect components of the ECS in the adipose tissue and uterus, which may be part of the immunometabolic effects of this FA. The expression of the enzyme diacylglycerol lipase A (DAGLA), which synthesizes the eCB 2-AG, was higher in adipose tissue of cows supplemented with CLA vs. controls, and the mRNA and protein abundances of CB1 were reduced in CLA supplemented cows (Daddam et al., 2021). In addition, CLA supplementation to transition dairy cows reduced the expression of uterine CNR2/CB2 and NAPEPLD (Abolghasemi et al., 2016). These findings indicate that CLA may exert some of its immunometabolic effects via modulation of the ECS.

### Niacin

Lipolysis is a process associated with enhanced production of oxilipids and other lipid mediators of inflammation. This is due to the activation of biosynthetic enzymes and the release of substrates (i.e., FAs). Thus, a logical intervention to reduce the risk for immunometabolic dysfunction is to inhibit lipolytic pathways or to promote lipogenic activity that re-esterifies FAs into triglycerides in the lipid droplets of adipocytes (Contreras et al., 2018).

Niacin as nicotinic acid is a well-known inhibitor of the activation of bovine hormone-sensitive lipase, the most active lipolytic enzyme during the periparturient period of dairy cows (Kenéz et al., 2014). Rumen-protected niacin is effective in reducing lipolysis in early lactation when fed during the entire transition period starting at least 3 wk before expected parturition and until 3 wk after calving (Yuan et al., 2012). In monogastrics, niacin modulates the inflammatory and metabolic functions of immune cells. The downstream product of niacin, nicotinamide, is an essential component of the metabolic pathways that are activated when macrophages shift from aerobic glycolysis—associated with M1 pro-inflammatory phenotype—to oxidative phosphorylation—a feature of the M2 pro-resolving phenotype (Suchard and Savulescu, 2022). The second mechanism of immunometabolic modulation of niacin is its direct effect on the oxylipid biosynthesis. Prolonged supplementation of niacin enhances the synthesis of omega-3 FAs and their anti-inflammatory oxylipid products (Heemskerk et al., 2014). The direct effects of niacin on dairy cows’ immunometabolism are sparsely described. However, since bovine adipocytes, mammary and uterine epithelial cells, hepatocytes, and immune cells, including macrophages, neutrophils, and lymphocytes express the niacin receptor GPR109A/HCA2 (Xiao et al., 2017), it is expected that these cells will respond favorably to the effects of niacin during immune challenges. Therefore, niacin could be considered as a potential immunomodulator in transition dairy cows.

## Conclusions

**Figure 4.**
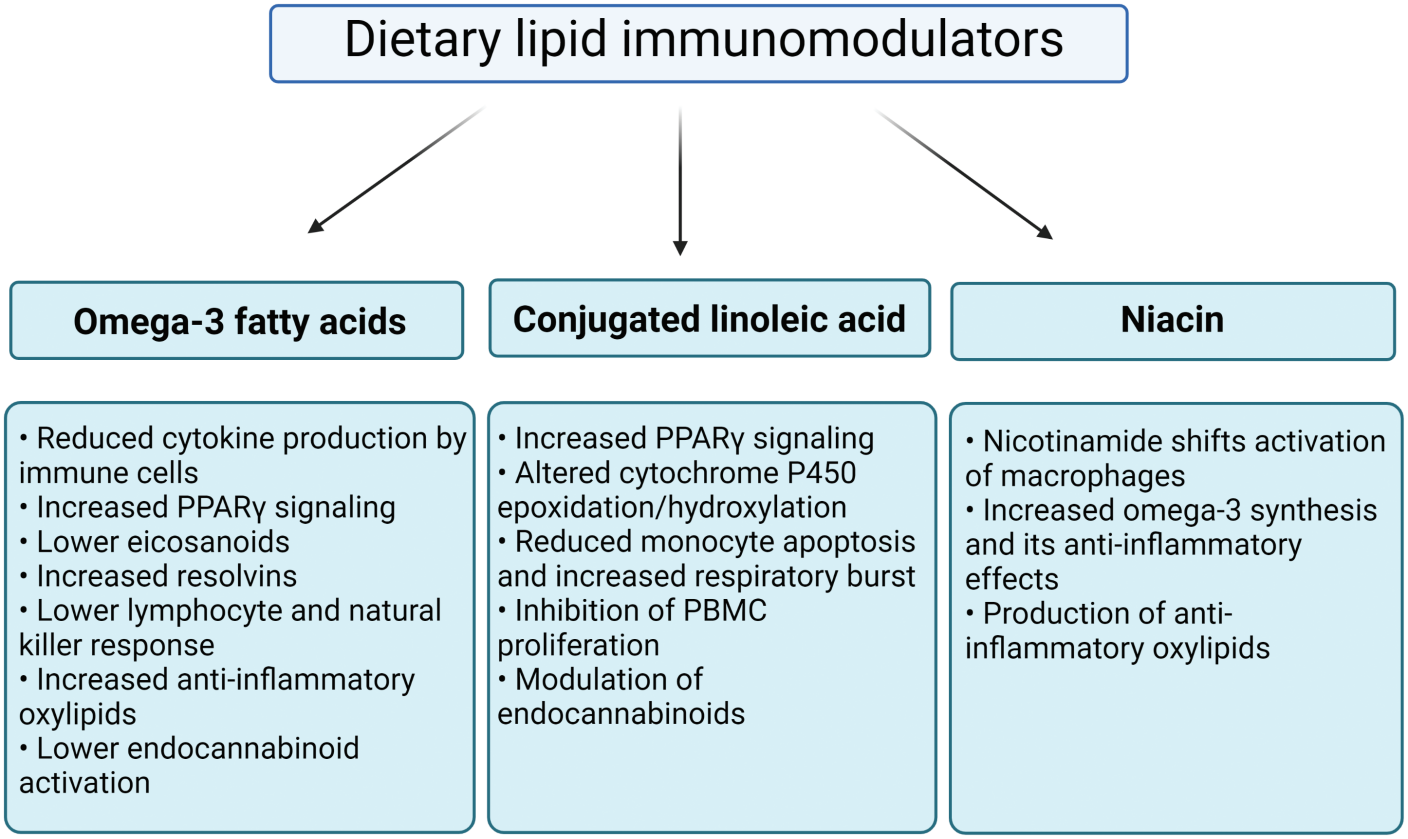
Dietary lipid immunomodulators. Supplementation of omega-3 FAs, conjugated linoleic acid (CLA), and niacin exert multiple effects on immune function and inflammation and can be used as nutritional strategies to modulate immunometabolism in transition dairy cows. Image was prepared using Biorender.com.

Immunometabolism is an important field of research in dairy cows, specifically during the transition period, as many changes in metabolic and immune function occur at this time. Here we highlighted lipid immunomodulators: eCBs, FAs, and oxylipids, that each one and the combination of them may affect the immunometabolic function of transition dairy cows. We described three dietary manipulations that be implemented to modulate immunometabolism; omega-3 and CLA FAs as well as niacin (Figure 4). Each of these strategies can affect inflammation, immune cell function, and oxylipid production. Moreover, omega-3 and CLA can also modulate the activation of the ECS, which is involved in the regulation of immune function. More research is required to further explore the mechanisms and outcomes of immunometabolic modulations in transition dairy cows.

## About the Authors

**Maya Zachut** is a research scientist at the Department of Ruminant Science, Institute of Animal Science, Agriculture Research Organization, Volcani Institute, Israel. She received her MSc and PhD in Animal and Veterinary Sciences from the Faculty of Agriculture, Food and Environmental Quality Sciences of the Hebrew University of Jerusalem, Israel. Zachut’ MSc and PhD were on the effects of dietary fatty acids on reproduction in dairy cows. Her postdoctoral training was at Weizmann Institute of Science and Volcani Institute, on the molecular mechanism of insulin resistance in transition cows. Zachut’ research focuses on improving the physiology of dairy cows during metabolic and heat stresses; by modulating inflammation and the endocannabinoid system. Zachut leads a collaboration with Professors Tam and Contreras on modulation of the endocannabinoid system in transition dairy cows. Corresponding author: mayak@volcani.agri.gov.il

**Joseph Tam** received his BMedSc, MSc, PhD, and DMD from the Hebrew University of Jerusalem. He did his postdoctoral training at the National Institutes of Health (NIH), and in 2014, Prof. Tam moved to the Hebrew University, where he heads the Obesity and Metabolism Laboratory at the Institute for Drug Research, and focuses on targeting the endocannabinoid (eCB) system for obesity, diabetes, and the metabolic syndrome. He also serves as the Director of the Hebrew University’s Multidisciplinary Center for Cannabinoid Research and a Scientific Advisory Board Member of several biotech companies, which develop a portfolio of non-psychoactive cannabinoid and cannabinoid-modulating medicines for unmet market needs. Having a clinical background with basic science training, Prof. Tam has always been interested in how science can directly improve people’s everyday lives. Thus, he has strived unceasingly to integrate his clinical curiosity and experimental knowledge to deepen the understanding of clinically relevant research questions.

**G. Andres Contreras** is a veterinarian and an associate professor at the College of Veterinary Medicine, Michigan State University (MSU). He practiced veterinary medicine in central Colombia working with tropical cow-calf operations and grazing dairy herds, and then in West Michigan servicing large dairy herds through the Large Dairy Internship Program at MSU. Contreras has a master’s degree in mastitis and milk quality and a PhD in Comparative Medicine and Integrative Biology. His postdoctoral training was at The Center for Integrative Metabolic and Endocrine Research (CIMER) at Wayne State University and focused on the lipolysis-induced white adipose tissue remodeling process and on elucidating the effects of sympathetic innervation on the differentiation of adipocytes in different fat depots. Contreras’ research program is focused on the adaptations of adipose tissue to disease and negative energy balance and its implications for dairy cattle health, especially during the periparturient period.
